# Lifestyle intervention and cognitive outcomes in Down syndrome: a horizon 21 European Down syndrome consortium scoping review

**DOI:** 10.1186/s11689-026-09694-0

**Published:** 2026-04-21

**Authors:** Eimear McGlinchey, Sarah Pape, Shahid H. Zaman, Jessica Eustace-Cook, Anna Stockbauer, Eleni Baldimtsi, Ellen Melbye Langballe, Frode Kibsgaard Larsen, Katja Sandkühler, Phoebe Ivain, Anne-Sophie Rebillat, Pierre Ecrement, Mary McCarron, Bessy Benejam, Wan Ming Khoo, Juan Fortea, Johannes Levin, Fredrik Öhman, Ann-Charlotte Granholm-Bentley, Andre Strydom, Georg Nübling

**Affiliations:** 1 Trinity Centre for Ageing and Intellectual Disability, Trinity College Dublin, Dublin, Ireland; 2 Global Brain Health Institute, Trinity College Dublin, Dublin, Ireland & University of California, San Francisco, California USA; 3 Department of Forensic and Neurodevelopmental Sciences, Institute of Psychiatry, Psychology & Neuroscience, King’s College London, London, UK; 4 Cambridge University, Cambridge, UK; 5 The Library of Trinity College Dublin, Trinity College Dublin, Dublin, Ireland; 6 Department of Neurology, Ludwig-Maximilians University, Munich, Germany; 7 German Center for Neurodegenerative Diseases (DZNE), Munich, Germany; 8 1st Department of Neurology, Medical School, Aristotle University of Thessaloniki, Thessaloniki, Greece; 9 The Norwegian National Centre for Ageing and Health, Vestfold Hospital Trust, Tønsberg, Norway; 10 Department of Geriatric Medicine, Oslo University Hospital, Oslo, Norway; 11 Institute of Clinical Medicine, Faculty of Medicine, University of Oslo, Oslo, Norway; 12 Institut Jérôme Lejeune, Paris, France; 13 Barcelona Down Medical Center, Fundació Catalana Síndrome de Down, Barcelona, Spain; 14 Sant Pau Memory Unit, Department of Neurology, Hospital de la Santa Creu i Sant Pau, Biomedical Research Institute Sant Pau, Universitat Autònoma de Barcelona, Barcelona, Spain; 15 Centro de Investigación Biomédica en Red en Enfermedades Neurodegenerativas (CIBERNED), Madrid, Spain; 16 Munich Cluster for Systems Neurology (SyNergy), Munich, Germany; 17 Department of Psychiatry and Neurochemistry, University of Gothenburg, Gothenburg, Sweden; 18 Center on Aging, Department of Neurosciences, Medical University of South Carolina, Charleston, SC USA

**Keywords:** Down syndrome, Cognitive decline, Lifestyle interventions, Non-pharmacological strategies, Dementia prevention, Modifiable risk factors, Alzheimer's disease

## Abstract

**Background:**

Life expectancy for individuals with Down syndrome (DS) has significantly increased, primarily due to medical advances. While DS is considered a genetically determined form of Alzheimer’s disease (DS-AD), with neuropathological markers evident by age 40, the onset of clinical dementia varies. Modifiable risk factors are thought to contribute meaningfully to dementia risk in the general population. Advances in intervention studies in the general population suggest cognitive decline can be reduced through multimodal lifestyle interventions, however no large-scale multimodal studies have been conducted in the DS population.

**Search strategy:**

A comprehensive search was conducted across five electronic databases—Medline, EMBASE, CINAHL, Web of Science, and ASSIA to identify studies that examined the relationship between lifestyle interventions and cognitive outcomes in adults with DS. The search combined database-specific controlled language with keywords related to exercise, diet, social activities, cardiovascular health, and brain stimulation. Studies included were peer-reviewed original research articles focusing on adults with DS and reported on cognitive outcomes or AD-related biomarkers.

**Results:**

The search yielded 24,774 articles, with 16,868 remaining after duplicates were removed. A total of 44 articles met inclusion criteria across the domains of exercise, diet, cardiovascular health, social connectedness, and cognitive stimulation. Most studies focused on exercise, indicating some cognitive benefits, particularly in executive functions and working memory, though results were inconsistent, and many suggested the necessity of high adherence to intervention protocols. No studies were found that examined the direct impact of diet on cognition in DS. Findings on cognitive stimulation, cardiovascular health and social connectedness suggested potential but inconclusive benefits for cognitive function.

**Conclusions:**

This review underscores the significant gaps in research regarding non-pharmacological interventions for DS-AD. It highlights the need for tailored, well-structured studies to better understand and leverage potential cognitive benefits of lifestyle interventions in the DS population. Implementing such interventions early in life and before significant disease progression may help maintain quality of life and independence among individuals with DS. Future research should focus on comprehensive, multi-domain interventions to ascertain their efficacy and optimal application.

**Supplementary Information:**

The online version contains supplementary material available at 10.1186/s11689-026-09694-0.

## Introduction

Life expectancy for people with Down syndrome (DS) has increased from 30 years in the 1930s [1] to over 60 years today [2]. DS is associated with accelerated ageing [3], and a distinct pattern of age-related co-occurring conditions compared to the general population [4], most notably, DS associated Alzheimer’s disease (DS-AD). DS is a genetically determined form of Alzheimer’s disease (AD) [5]. Almost everyone with DS develops neuropathological hallmarks of AD by age 40 [5, 6] with an associated risk greater than 95% of clinical dementia by the seventh decade [7, 8]. Variability in the age of onset of symptoms [9], despite a common genetic predisposition and a predictable sequence of biomarker changes [8], suggest that other factors may influence the relationship between neuropathology and cognitive symptomatology. In the general population, the 2024 Lancet Commission identified 14 modifiable risk factors estimated to reduce dementia risk worldwide by approximately 50% [10], though the modelling underpinning these estimates relies on assumptions that may not translate directly to intervention effects. Nevertheless, even a modest reduction in modifiable risk factors could delay onset, and this framework provides a valuable basis for identifying intervention targets, including in populations with DS, where the specific risk profile may differ substantially.

Recent advancements in non-pharmacological interventions have shown effectiveness in reducing cognitive decline, notably the Finnish Geriatric Study (FINGER), which applied a multidomain program in 1260 participants aged 60–77 [11]. Following a two-year intervention, positive benefits were shown for cognition, with a 25% improvement on neuropsychological test batteries, as well as reduced risk of chronic conditions, functional decline and overall improved health-related quality of life [12]. This study has since been implemented internationally across over 45 countries, with several variations including most recently a combination of multimodal lifestyle interventions with medication [13]. Such an intervention has not yet been conducted in a population with DS.

The aim of this scoping review is to examine and synthesise the available evidence on the association between the modifiable factors included in the FINGER study (exercise, diet, social activities, cardiovascular disease and cognitive stimulation) and cognition in people with DS. We will outline both gaps in the literature as well as opportunities for future research efforts.

## Methods

### Search strategy and selection criteria

Separate searches were run for each strand of interest (Exercise, Cognitive stimulation, Social Connectedness, Diet and Cardiovascular health), with ‘Down syndrome’ included as population and ‘cognition’ and ‘biomarkers’ as primary and secondary outcomes of interest in all searches. Database thesauri were reviewed for controlled language and synonyms. A keyword list was developed and adapted with additional input from co-authors. The search utilized a combination of database specific control language and keywords, which were combined with the OR Boolean operand (See Appendix 1 for search protocol for each strand). Five electronic databases were searched between November 5 and November 17, 2024: Medline, EMBASE, CINAHL, Web of Science and ASSIA. The search was re-run between 19 and 21 February 2026 to identify new and up to date articles. No limiters were added for time, language or geography. Publication format was limited to peer-review original research journal articles. Studies were included if participants included adults (18 + years) with DS, data on cognition and/or AD related biomarkers, and the domain of interest in each strand (see Appendix 2). Articles were imported into Covidence for screening, and duplicates were removed. Three authors (EMG, SP, GN) screened all articles for titles and abstracts. Full texts were assessed for eligibility by two authors with any disagreements resolved through discussion among the researchers. A qualitative synthesis was conducted to report on findings related to all strands impacting cognition and biomarkers in adults with DS.

## Results

The search yielded 24,774 articles across all domains, of which 16,868 remained after removing duplicates (Exercise (1,060), Cognitive stimulation (145), Social Connectedness (6,018), Diet (6501), Cardiovascular (3144)) (See PRISMA diagrams in Appendix 2). Following title, abstract and full text screening, a total of 44 articles were included in this review across 5 strands: Exercise (20), Diet (0), Cardiovascular (14) Social Connectedness (6), Cognitive stimulation (4). See Table 1 for summary of articles included. All studies were from high income countries, with 28 studies from USA, 6 studies from UK, 2 studies from Italy, and 1 study each from the remaining countries (Ireland, Australia, France, Greece, Netherlands, Spain, Taiwan, Portugal).

**Table 1:** Summary tables across all domains

Study	Single/repeated	Study design	*N*	Population	Dementia y/*n*	Activity / activity measure	Outcome measures	Outcome
EXERCISE								
Ringenbach 2016 [26]	8 weeks	Interventional	44	18+-4	No dementia	Assisted cycling therapy, voluntary cycling	Knock tap (NEPSY), Word lists (category, first letter), Wisconsin card sorting test (MCST), Reaction time: Visual Choice Reaction Time,	Improvement in reaction time, inhibitory control and semantic fluency
				W19:25 M				
Ringenbach 2021 [20]	Single time	Interventional	14	26+-5y	No dementia	Resistance training	TOL, Ericksen Flankner Task	Improvement in inhibition in RT and ACT, improvement in cogn. Planning in control and ACT
				F6:8 M		Assisted cycling therapy		
Chen 2014	n.a.	Cross-sectional / observational	12	19+-4	No dementia	PAL	Purdue pegboard, Corsi-block (visual working memory), auditory working memory span, TOL	PAL not associated with cognitive measures; fine motor dexterity associated with cognitive measures
Chen 2015 [21]	Single time	Interventional	20	22+-5	No dementia	Treadmill	Knock-Tap test, choice-response time (visual cue), DCCS	Improvement in inhibition after exercise
Chen 2016 [22]	Single time	Interventional	18	22+-4	No dementia	Treadmill, two intensity groups	Knock-Tap test, choice-response time (visual cue), DCCS	
Chen 2019 [23]	Single time	Interventional	28	22+-6	No dementia	Treadmill, two intensity groups	Word lists (categories, first letters)	Improvement in semantic, not phonematic verbal fluency upon moderate, not high intensity exercise
				14–31				
Ptomey 2018 [27]	12 week	Interventional	27	28+-8 18–35 W11:16 M	No dementia	Instructed exercise, controlled by fitBit	CANTAB: Attention switching task (AST), Paired Associates Learning (PAL), reaction time	Improvement in memory function with higher exercise
Fleming 2021 [16]	n.a.	Observational	61	37+-8	55 no dementia	7 day actigraphy measurement	CRT (cued recall test), Purdue Peg Board, WISC-IV/Haxby, Stroop Cats&Dogs	Better performance in visuospatial and executive function in more active participants
				W33:28 M	6 MCI		VMI (Developmental Test of Visual-Motor integration)	
Post 2022 [28]	10 weeks	Interventional	11	26+-6	No dementia	Resistance taining	CANTAB, Scales of Independent Behavior – Revised (SIBR)	Improvement in one task involving singular visual working memory
Holzapfel 2016 [30]	8 weeks	Interventional	44	18+-4	No dementia	Assisted cycling therapy, voluntary cycling	Wechsler Memory Scale III verbal memory digit span and reverse digit span	Improvement in verbal working memory, but not in verbal short-term memory in the ACT group
				W19:25 M				
Peven 2022 [17]	n.a.	observational	71	38+-8	4 MCI	4 day actigraphy measurement	Modified Cued	Higher memory function in those with more moderate physical activity. Better memory function in those with higher hippocampal volume, but activity and hippocampal volume were not associated
				W40:41 M	1 DS-AD		Recall Test (CRT) and the Rivermead Picture Recognition Test	
Shields 2022 [31]	12 weeks	Interventional		24+-7 13–35 W9:11 M	No dementia	Mentor-based exercise program	Executive functioning (planning,	Improvement only in BRIEF (global executive function), but not TOL or CS-DS
							response inhibition, attention shifting):	
							· TOL	
							· Sustained Attention to	
							· Response Task	
							· CANTAB Intra-extra Dimensional Set Shift Test	
							· Cognitive Scale for Down Syndrome,	
							· Behaviour Rating Inventory of Executive Function	
							· (BRIEF).	
							Working memory:	
							· CANTAB Paired Associates Learning task	
							information processing speed:	
							· Motor Screening Task	
Holzapfel 2015 [29]	8 weeks	Interventional	48	18+-4	No dementia	Assisted cycling therapy, voluntary cycling	Purdue peg board	Improvement in motor planning/Control
Perrot et al., 2021 [32]	12 weeks	Intervention	12	35+ (mean age = 50.35)	No dementia	Wii based exercise program	Corsi block tapping test	Improvement in physical and functional outcomes No significant differences in cognitive outcomes
Pape et al., 2021 [15]	2 year follow up	Longitudinal cohort study	214 baseline 91 at follow up	16+	No dementia	Classified as high, moderate, low reported by participants	CAMDEX-DS	Moderate and high intensity associated with reduced risk of decline memory and orientation High intensity linked to reduced risk in decline in personality and behaviour
Kenshole et al., 2017 [14]	10 year	Prospective cohort	57	46–78	27 with dementia 30 without dementia	Classified as high, moderate, low reported by participants	Onset of dementia	No difference between clinical and control sample
Merzbach et al., 2023 [25]	8 weeks	intervention	83	27	No dementia	Walking/jogging (3 × 30 min/week), cognitive training (~ 20 min/session, 6 times/week), or both.	Physical fitness (6-minute walk test), cognitive function (Corsi block test, SART, Stroop task).	Physical exercise alone led moderate cognitive improvements in tasks related to vigilance and decision-making (SART, Stroop task). Combined exercise and cognitive training resulted in the most comprehensive improvements, enhancing both physical fitness and cognitive function, with greater gains in selective attention, decision-making, and information processing.
Ringenbach 2025 [24]	8 weeks	Interventional	24	26 + yrs, mean age 37, older adults with DS	No dementia	Assisted cycling therapy (ACT) vs. voluntary cycling (VC), 30 min, 3x/week	TOL (cognitive planning), modified Wisconsin Card Sorting Test (set switching), Corsi Block Test (spatial memory)	Improvement in cognitive planning (TOL total correct) for both ACT and VC (*p* = 0.021). No significant differences for spatial memory or set switching
Fleming 2025 [18]	3.29 yrs follow-up	Longitudinal observational	69	26–58 yrs, mean age 39+-8.5, F36:M33	63 cognitively stable, 4 MCI, 2 dementia	7-day actigraphy (MVPA, min/day)	mCRT (episodic memory), DLD SOC and SOS (dementia symptoms), PET Aβ (centiloids)	MVPA not associated with baseline Aβ or change in Aβ (resistance). MVPA moderated association between Aβ increase and cognitive decline (resilience): higher MVPA associated with less decline on mCRT (*p* = 0.029) and DLD SOC (*p* = 0.034)
Clina 2025 [34]	n.a.	Cross-sectional	40	18–45 yrs, mean age 26+-7.8, 58% female	No dementia	Accelerometer (MVPA, 7 days), maximal treadmill test (VO2 Peak)	rsfMRI DMN functional connectivity (PCC seed), MVPA, VO2 Peak	VO2 Peak associated with overall DMN connectivity (*r* = 0.472, *p* = 0.004) and medial prefrontal cortex connectivity (*r* = 0.431, *p* = 0.010), significant after adjusting for age and sex. No association between MVPA and DMN connectivity
SOCIAL CONNECTIONS								
Mihaila et al., 2019 [39]	3 year follow up	Prospective cohort	65 baseline, 54 follow up	30–53	No demenita	Leisure activity questionniare	SB5, Cued Recall Test, Rivermead Behavioral Memory Test for Children. Brain β-amyloid using PET	Leisure activity with less decline in episodic memory. No difference in β-amyloid accumulation
Skotko et al., 2023 [37]	Single time	Prospective cohort	38	25–55	No dementia	N/A	PERSNET personal networks instrument	Personal networks in DS can be quantitatively analysed with no self-report/proxy differences
Brown et al., 1990 [35]	Single time	Retrospective cohort	130	1–59	No dementia		Stanford-Binet Test, Wechler Scales, Bayleys or Cattells, Slossons and Leiters.	Residential environment had impact on functional decline but not cognitive decline
Harisinghani et al., 2023 [36]	1 year follow up	Prospective cohort study	24	25+	No dementia	N/A	PERSNET personal network instruments.	Social network remained consistent despite external factors
Mihaila et al., 2020 [38]	Single time	Prospective cohort study	44	25–56	No dementia	Leisure activity diary	DLD	People with DS primarily initiated their own leisure activities, but limited by transportation needs.
Schworer 2025 [40]	n.a.	Cross-sectional	63	28–59 yrs, mean age 40+-7.7, ABC-DS cohort	84.2% cognitively stable, 9.5% MCI, 6.3% dementia	Lifestyle composite: leisure activity frequency, employment activity frequency, physical activity (actigraphy step count)	NTG-EDSD (dementia symptoms), DSMSE (cognition), PET Aβ (amyloid age)	Significant moderation effect of lifestyle composite on association between amyloid age and NTG-EDSD and DSMSE. Higher lifestyle composite (more leisure, employment, PA) associated with fewer dementia symptoms given similar amyloid burden
COGNITIVE STIMULATION								
McGlinchey 2019 [41]	8 week follow up	Intervention	40	30–49	No dementia	Computerised cognitive training	Objective Measures of executive function: Cats and Dogs Tower of London Scrambled Boxes Spatial Reversal Weigl Card Sorting Proxy Measures: BRIEF- A	Improvement in Cats and Dogs, Tower of London No transfer to everyday life
Ali et al., 2022 [43]	20 week follow up	RCT	23	40+	Dementia	Individual cognitive stimulation therapy	CAMCOG-DS and Modified Memory for Objects test. Proxy Measures: The CSDS, ADCS-ADL and QOL-AD	No change in cognition or adaptive functioning Increase in quality of life
Anagnostopoulou et al., 2021 [42]		Intervention	12	29 ± 11 years	No dementia	Computerized physical training (PT) and cognitive training (CT)	Cognitive measures: WISC III (Digits Span, Picture Arrangement, Block Design, Mazes) Raven Reading the mind in the eyes Somatometric measures, including tests to appraise functioning mobility, flexibility, dynamic stability, strength and balance	Combined PT and CT in adults with DS can trigger neuroplasticity resulting in cortical reorganization.
deLaTorre et al., 2016 [44]	12 month follow up	RCT	84	16–34	No demenita	Epigallocatechin-3-gallate (EGCG)+cognitive training vs. placebo+cognitive training	Measures of attention, psychomotor speed, memory, executive functions, language, adaptive behaviour, quality of life, quality of sleep and neuropsychiatric symptoms were included.	EGCG + cognitive training improved memory and executive functions, which were accompanied by improved performance on daily tasks requiring basic literacy.
CARDIOVASCULAR								
Artal at al., 2017 [47]	Single	Retrospective cohort	13	Mean age 45.8	No dementia and dementia	prevalence of lifestyle related health factors (type 2 diabetes, hypothyroidism, lipid profile) and their potential associations with AD.	Dementia diagnosis	Individuals with DS and AD have a higher prevalence of metabolic risk factors
Vetra no et al., 20216 [48]	Single	Cross sectional cohort	36	18 + mean age 36.1	No dementia	Association between diastolic dysfunction and intellectual disability	Raven’s Matrice + Wechsler Adult Intelligence Scale	prevalence of diastolic dysfunction in Down Syndrome, associated with poorer cognitive scores.
Cooper et al., 2016 [57]	12 month	RCT	21	50 + Mean age 54	No dementia	Simvastatine 40 mg daily at night compared with a placebo	NADIID battery Selective Attention Cancellation Test Pattern recognition memory from the Cambridge Neuropsychological Test Automated Battery (CANTAB) Cats and Dogs test Tower of London Test Cued Recall Test Category Fluency Test Story Recall Test	Positive effect on NADIID with improving scores in simvastatine group and decreasing scores in placebo group Mixed effect of simvastatine for others cognitive measures, none statistically significant
Dodd et al., 2023 [49]	Single	Cross sectional cohort	79	18 + Mean age 26.7	No dementia	Association between BMI and cardiorespiratory fitness	Cambridge Neuropsychological Test Automated Battery for DS (CANTAB DS) = > Multi tasking, paired associated learning, reaction time	increased BMI is associated with decreased cardiorespiratory fitness, but isn’t associated with cognition and physical activity.
Huang et al., 2023 [50]		Cohort	32,783	24,5 +/- 15	No dementia	Assessing 10 years incidence of cardiac, renal and urinary tract complications in DS vs. matched controls		Significant higher risks of ischemic heart disease ; hypertensive disease and CKD
Lai et al., 2021 [51]	Single	Retrospective Cohort	339	Median 55	125 cognitively stable 214 possible/probable Alzheimer disease	Inflammatory conditions	AD	No association between AD and inflammatory conditions
Patel et al./, 2004 [52]	Single	Retrospective Cohort	116	Mena age 51.2	No dementia	BMI	Selective Reminding Test Down Syndrome Mental Status Examination (DSMSE) Block Design sub test (fro WISC-R) Extended block design (DSMSE) Beery Visual Motor Integration McCarthy verbal fluency	Among DS post menopausal women, obesity is associated with higher levels of estrone and better performances in some cognitive tests.
Percy et al., 2020 [53]	Single	Retrospective Cohort	29	Mean age 45.7	No dementia		Dementia Test Battery (DTB) including : Learning Memory Test (Dalton/McMurray visual memory test) Praxis test Multi Dimensional Observation Scale for Elderly Subjects Adapted for Persons with Down Syndrome (fives sub scales for physical, cognitive, emotional and social behaviours)	DTB scores correlates moderately with age, more pronounced among patients with heart disease. Low DTB scores (lower than 1 SD below 0) are significantly associated with heart disease, especially among patients with hypothyroidism
Prasher et al., 2008 [54]	Single	Retrospective Cohort	179	38–53 Median age 45	No dementia	Investigate the relationship between serum total cholesterol (TC), ApoE and Alzheimer Disease (AD)	AD	No statistically significant relationship between serum TC levels and AD in DS patients. Patients with DS and ApoE4 allele have higher TC levels.
Rosser et al., 2018 [55]		Prospective Cohort	234	6–25 Mean age 13,49 +/- 4,53 yo	No dementia	relationship between congenital heart defects (CHD) or gastro intestinal defects requiring surgery in the first year of life and intellectual disability among patients with DS	Arizona Cognitive Test Battery (including many sub tests measuring memory, executive functions and motor skills)	Having and ASVD or a GI structural defect does not significantly predict worse cognitive or behavioural outcomes in school age children with DS
Startin et al., 2020 [56]			602	: 4 sub groups (Younger children 0–5,5 yo ; older children 5,5–15 yo ; younger adults 16–35 yo ; older adults > 36 yo) ; 3,6 months old – 73 yo	No dementia	- Explore relationships between receptive language ability and general cognitive abilities with age and health comorbidities (including congenital heart defects, obstructive sleep apnoea, diabetes)		In younger adults, socio economic status, autism and epilepsy contributed to variance in cognitive ability
Frank et al. (2023) [58]	Single	Cross sectional	72	Adults. Mean age 26.8	No dementia	Cardiorespiratory fitness (VO2 Peak), systolic blood pressure, moderate to vigorous physical activity (MVPA).	Cognition (CANTAB DS battery measuring multitasking, episodic memory, reaction time).	Cardiorespiratory fitness correlated with simple movement time; systolic blood pressure was associated with episodic memory and reaction time. No association between MVPA and cognitive outcomes.
Brothers 2025 [65]	n.a.	Cross-sectional	262	Mean age 44+-9.3, ABC-DS cohort	84.2% cognitively stable, 9.5% MCI, 6.3% dementia	Cardiovascular risk composite (Framingham: BMI ≥ 30, SBP ≥ 130, statins, hypertension, hyperlipidemia, diabetes)	mCRT, DSMSE, PET Aβ (centiloids), resistance and resilience scores	Higher cardiovascular composite associated with resistance to Aβ (*r* = 0.156, *p* = 0.016). Hyperlipidemia associated with 1.8x likelihood of greater than expected Aβ for age (OR = 1.83, *p* = 0.047). No significant association with resilience scores
Clina 2026 [59]	n.a.	Cross-sectional (age/sex matched)	164 (82 CHD, 82 no CHD)	Mean age 40+-8.3, range 26–63, ABC-DS cohort	87.2% no MCI/dementia, 4.3% MCI, 6.1% dementia	CHD status (present/absent), surgical repair history	mCRT, Stroop, DSMSE, DLD, WISC-IV Block Design and Haxby Extension, PET Aβ (centiloids)	CHD group scored lower on visuospatial ability (β=-3.515, *p* = 0.022). CHD group had higher centiloid load (39.8 vs. 29.8, β = 8.00, *p* = 0.036) and projected to reach Aβ positivity ~ 4.5 yrs earlier (37.6 vs. 42.1 yrs). No other cognitive differences

**Fig. 1:**
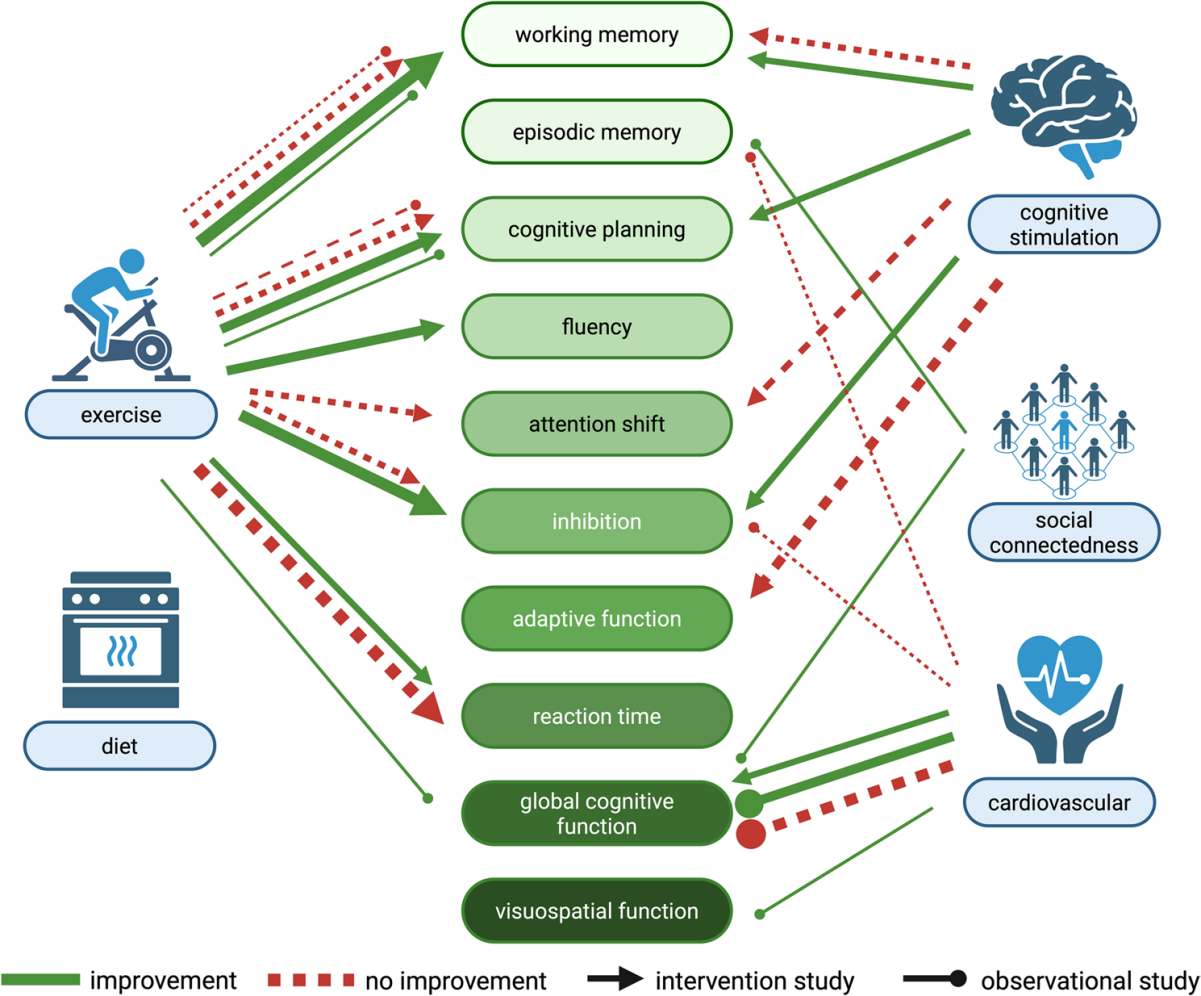
Positive and negative associations between domains of interest and cognitive domains. Legend: Associations Between Domains of Interest and Cognitive Domains: This figure illustrates the relationships between various cognitive domains and five key areas of interest: exercise, diet, cognitive stimulation, social connectedness, and cardiovascular health. Green arrows represent studies where an association was found, while red arrows indicate studies where no association was observed. Different arrow types distinguish between observational studies and intervention studies. The thickness of the arrows corresponds to the number of studies, with thicker arrows indicating a greater number of studies

Figure 1 provides a visual summary of the positive and negative associations found between the domains of interest (exercise, diet, cognitive stimulation, social connectedness, and cardiovascular health) and various cognitive domains.

### Exercise

The majority of non-pharmacologic studies in the review focused on exercise (*n* = 20). No association between exercise intensity and dementia status was found, although very low levels of physical activity noted for all participants [14]. Pape et al. [15] in their longitudinal cohort study, found that moderate and high-intensity exercise was associated with a reduced risk of decline in memory and orientation, with high intensity exercise also associated with a reduced risk of decline in the personality and behavior domain.

Two observational studies in the Alzheimer Biomarker Consortium-DS (ABC-DS) cohort established that higher everyday physical activity was associated with improved performance in several cognitive domains including memory, visuospatial and executive function [16, 17]. These two studies did not find an association between physical activity and DS-AD biomarkers (Amyloid and Tau PET, hippocampal volume). However, a subsequent longitudinal analysis within the same cohort (Fleming et al., 2025) found that while MVPA did not predict changes in amyloid burden over approximately three years, it did moderate the relationship between increasing amyloid and cognitive decline, with higher MVPA associated with less decline on the mCRT and fewer dementia symptoms, suggesting a resilience rather than resistance mechanism [18]. Previous studies have suggested that physical activity promotes compensatory cognitive mechanisms in DS-AD, at least prior to dementia onset, however, it is also possible that people with better cognitive function simply tend to be more active or face fewer barriers to participate in physical activity [19].

A number of small interventional studies investigated the effect of single exercise bouts on selected cognitive domains (see Table 1). These studies by Chen and Ringenbach [20–23] most consistently found improvements in inhibitory function as well as cognitive planning, semantic fluency and choice reaction time in singular studies, indicating susceptibility of executive function domains to activation. A further study by the same group in older adults with DS (mean age 37) confirmed that both assisted cycling therapy and voluntary cycling improved cognitive planning over eight weeks, though not spatial memory or set switching [24]. The recent MinDSets study found that eight weeks of prescribed exercise improved attention and decision-making in adults with DS, with even greater cognitive benefits observed when combined with cognitive training [25]. In total, we identified seven publications [26–32] reporting on five longitudinal interventions, which included assisted cycling therapy and instructed exercise programs from eight to twelve weeks. These studies reported inconsistent results, with positive effects on visual working memory found in two out of three interventions, and an additional positive finding on auditory working memory in the fourth intervention. Singular positive results were reported on motor planning and semantic verbal fluency, while attention shifting, and reaction time were mostly unaffected. Importantly, therapy adherence was high in these studies, indicating that engaging in up to two exercise units and up to 30 min per week is feasible, at least in the younger DS population. Most studies including a control group found learning effects within the control group, which should be taken into account for sample size calculation, while also recognizing that learning effects themselves may be a measure of improved cognition. An additional study by Perrot et al. [33] conducted a 12-week Wii-based exercise program with adults over 35 years of age, which improved physical and functional outcomes but not cognitive outcomes, but noted that exercise intensity was below moderate as measured by heart rate. Emerging evidence also suggests that cardiorespiratory fitness specifically, rather than physical activity volume alone, may be relevant to brain health in DS. Clina et al. (2025) found that VO2 Peak was associated with resting-state functional connectivity of the default mode network in adults with DS, whereas MVPA was not, highlighting the potential importance of fitness-based targets for intervention [34].

Overall, current evidence indicates that exercise may positively impact singular cognitive domains, but with little evidence of how this relates to meaningful change in clinical function. A careful selection of the investigated domains is warranted, as some may not respond to exercise. Additionally, using global/composite scores as primary endpoints should be approached with caution. Despite exercise being the most studied domain, evidence of its effect on cognition in people with DS remains sparse and inconsistent.

### Diet

To our knowledge, no studies relating diet to cognitive decline have been conducted in this population.

### Social connectedness

Five prospective and one retrospective study examined social connectedness in people with DS. Brown et al. [35] explored the impact of residential setting on intellectual and adaptive function over time. While cognitive decline was consistent across environments, adaptive skills deteriorated less in institutional settings. This may suggest that the structured environment in institutional settings help preserve adaptive skills, while a more supportive atmosphere in home settings, perhaps with aging parents, may contribute to a greater decline in these abilities [35]. Social networks of people with DS remained stable over time without external stimulation, suggesting resilience in social connectivity [36, 37], and leisure activity is typically initiated by either the person with DS or someone from family or support staff [38]. One prospective cohort study (*n* = 65) found that higher engagement in social leisure activity was associated with less decline in episodic memory, but with no differences in accumulation of β-amyloid [39]. It is thus conceivable that an externally motivated increase in social connectedness may decrease progression of symptoms in a compensatory manner. Supporting this, Schworer et al. (2025) found that a lifestyle composite incorporating leisure activity, employment engagement, and physical activity significantly moderated the association between amyloid burden and dementia symptoms in 63 adults with DS, with higher lifestyle engagement associated with fewer symptoms at a similar level of amyloid pathology [40].

### Cognitive stimulation

Four studies on cognitive stimulation were included in the review. The interventions varied significantly in content (cognitive training, physical training, individual cognitive stimulation therapy (iCST), EGCG (epigallocatechin-3-gallate supplementation) and duration (ranging from 8 weeks to 12 months) and sample sizes (number of participants ranging from 12 to 80). The studies employed a range of outcome measures (See Table 1), from objective and proxy measures of executive function to cognitive measures, quality of life assessments, and neurophysiological changes. One study found improvements in some domains of executive function after an 8 week intervention in middle aged individuals without dementia [41]. A small pilot study in 12 younger adults (mean age 29 years) reported improved cognitive performance after a 10-week combined physical and cognitive training intervention, but could not rule out learning effects in the cognitive tests due to lack of a control group [42]. Nonetheless, this study also demonstrated improved functional network connectivity indicating a beneficial effect. Ali et al. [43] reported no significant differences in cognitive and adaptive functioning following iCST, although quality of life improvements were noted. A larger randomized controlled study (*n* = 84) primarily evaluated the effect of ECGC on cognitive performance in DS [44]. This 12-month phase two trial did not show cognitive improvement in the control group, which only received cognitive training. It also highlighted that adherence to the extensive digital cognitive training regimen (45 min, three times a week) was suboptimal over a prolonged time period. It may be necessary to ensure caregiver engagement in cognitive training programs and to reduce training time in future cognitive interventions. Overall, studies have shown that cognitive training interventions are feasible both as manual-based carer-delivered interventions and as digital interventions via an online platform [41, 43], but evidence of efficacy was varied overall. Furthermore, while some studies observed improvements in specific cognitive or physical capacities, there was a notable challenge in translating these gains into everyday functional improvements.

### Cardiovascular risk factors

Given the high comorbidity of vascular dementia and AD in the general population as well as its association with cardiovascular risk factors, modifying these factors was identified as one of the key pillars in FINGER trials. In contrast, while vascular cerebral damage in individuals with DS can be detected as early as age 30, it appears to be mostly due to cerebral amyloid angiopathy (CAA) [45, 46], although the impact of CAA on cognitive decline in dementia is not fully understood. It is therefore important to carefully evaluate the potential effects of cardiovascular disease on DS-AD and cognition in people with DS.

Fourteen studies examined the association between cardiovascular health status and cognitive outcomes in people with DS. The observational studies primarily assessed associations between various cardiovascular and metabolic health factors (e.g. congenital heart disease, thyroid function, lipid profiles, and diastolic dysfunction) and cognitive outcomes [47–56]. One intervention study exploring the effects of Simvastatin, a cholesterol lowering drug, on cognitive functions found that Aβ40 levels changed less for the simvastatin group, but this was not statistically significant [57]. A recent study found that cardiorespiratory fitness and systolic blood pressure were associated with aspects of cognition such as episodic memory and reaction time, in adults with DS, highlighting the potential impact to cognitive outcomes in this population [58].

Several studies have shown that the presence of congenital heart disease does not influence cognitive performance in children and adolescents with DS [55, 56]. In contrast, two small studies suggested that cardiovascular disease may impact cognition in older adults with DS [48, 53]. A larger age- and sex-matched analysis within the ABC-DS cohort found that adults with congenital heart disease had higher amyloid burden and were projected to reach amyloid positivity approximately 4.5 years earlier than those without, and scored lower on visuospatial ability, though no other cognitive differences were observed [59]. Similarly, while people with DS demonstrated alterations of lipid metabolism in some, but not all studies, which appear to be susceptible to exercise [60], Brothers et al. (2025) found that a cardiovascular risk composite was associated with greater-than-expected amyloid accumulation for age in 262 adults with DS, with hyperlipidemia specifically conferring a 1.8-fold increased likelihood of earlier amyloid positivity.

One possible target for a FINGER-like intervention may be atrial fibrillation. It was shown that people with DS are at increased risk of stroke [61]. At the same time, atrial fibrillation appears to be more frequent in people with DS, possibly due to congenital cardiac conditions, and is associated with an increased risk for cerebrovascular events [61, 62]. A multimodal intervention in DS should therefore potentially focus on the early detection of atrial fibrillation, and early intervention of preventing embolic stroke.

In summary, while cardiovascular risk has historically been less explored in relation to cognitive decline in DS than in the general population, recent evidence from larger cohorts suggests that cardiovascular conditions may influence the timing and trajectory of AD pathology in DS, warranting further investigation.

## Discussion

Applying an intervention developed for the general population to individuals with DS requires adaptation to meet specific risk factors and needs of this population. The majority of people with DS have mild to moderate intellectual disability (75–80%) with comorbid conditions such as epilepsy and autistic spectrum conditions additionally affecting cognitive outcomes [56, 63]. Although level of intellectual disability does not appear to correlate with the age of dementia onset [64], it does influence the level of support required for effective interventions. Moreover, compliance may present unique challenges, with different factors influencing the adherence to the intervention than seen in the general population. Understanding the reasons for both compliance and non-compliance is essential, and feasibility studies should focus on identifying these factors to ensure practical and effective implementation. This may involve engaging both caregivers and individuals with DS in the development of protocols to identify and address potential barriers. The limited evidence available already points towards a different weighing of the FINGER domains in the DS population. While exercise, cognitive stimulation and social interaction may be promising targets for intervention, cardiovascular health interventions need to be adapted to suit the largely different incidences of disorders in this domain. For example, more people with DS will require monitoring and treatment of OSA, diabetes, thyroid disease and obesity than would occur in the general population. Recent evidence that congenital heart disease may accelerate amyloid accumulation [59] and that cardiovascular risk factors are associated with earlier amyloid positivity [65] further underscores the need to address cardiovascular health within any multimodal intervention for this population.

The person’s age at intervention should also be considered. The average age of onset of dementia in people with DS is 53.8 years [9], with a predictable sequence of biomarker changes starting approximately two decades earlier [8], meaning that interventions should start earlier than in the general population. Support from caregivers is critical, and their ability to motivate and adhere to the intervention’s protocols is likely to impact its success. This might necessitate specialized training (train the trainer) or additional motivation for caregivers. Furthermore, the individual’s place of residence and transportation needs must be taken into account when implementing interventions [38]. For example, in trials such as the original FINGER study and its extensions (Met-FINGER, U.S. POINTER), social engagement was promoted through regular group meetings and encouraged interactions both during and outside of these sessions [12, 13, 66]. Implementation of this with a population with DS could look different depending on service delivery. Finally, to ensure sufficient participant numbers and comprehensive delivery, interventions should adopt a collaborative multinational approach. This necessitates consideration of international service delivery variations and flexibility within the intervention protocols to accommodate these differences. Emerging evidence suggests that the combined effect of multiple lifestyle factors may be greater than any single domain in isolation. Schworer et al. (2025) found that a composite of leisure activity, employment engagement, and physical activity moderated the association between amyloid burden and dementia symptoms, with higher lifestyle engagement associated with better cognitive outcomes at similar levels of pathology. This provides preliminary support for the multimodal approach advocated by the FINGER framework and suggests that such an approach may confer resilience to AD pathology in DS.

While the focus of this review has been on cognitive outcomes, lifestyle interventions are also likely to benefit quality of life, social participation, and independence; outcomes that are of central importance to people with DS and their families. Future intervention studies should consider incorporating quality of life as a key outcome measure alongside cognition.

### Potential targets for intervention beyond FINGER

While not included in the FINGER study, the following domains could be appropriate for a lifestyle intervention study including people with DS.

#### Sleep

In people with DS there is a high prevalence of sleep disorders, especially obstructive sleep apnea (OSA). This is in part secondary to macroglossia and retrognathia as well as an increased frequency of obesity in young adulthood. Observed frequencies of OSA in adults vary mostly depending on methodology, with studies applying polygraphy or polysomnography yielding OSA prevalence of various severity between 42% and 94%, with severe OSA being present in up to 44% of patients. Importantly, it was shown that OSA can be associated with impairment in certain cognitive domains such as visual perception or executive function [67–69]. Moreover, OSA was associated with increased white matter lesions and overall amyloid burden in a cohort of older people with DS (mean age 50 years) [70]. Thus, actively searching for OSA appears to be a warranted target for a lifestyle intervention trial in DS, preferably via polygraphy or polysomnography since self-reported measures may miss out on OSA in this population [71, 72].

#### Endocrinologic, metabolic and hematologic disorders

DS is associated with a wide variety of systemic disorders. With respect to cognition, attention should be directed towards the highly prevalent thyroid dysfunction, but also other factors such as anemia or vitamin disorders. DS is associated with a high incidence of thyroid dysfunction, particularly hypothyroidism, which has been associated with poorer cognitive outcomes in the general population. People with DS are also more likely to develop diabetes mellitus, with children and young adults up to 4 times more likely to be diagnosed than the general population [73]. Diabetes has been linked to poor vascular health, and is a contributor to both vascular and Alzheimer’s dementia [74].

#### Sensory disorders

Visual and hearing disorders are highly prevalent within the DS community. Vision impairment can occur in up to 80% of the older DS population [75] due to various reasons such as refractive errors, cataracts or keratoconus. People with DS also have narrow auditory canal, which can lead to wax build-up which can contribute to hearing loss [76]. Furthermore, age-related sensorineural hearing loss can occur before the age of 30 [77]. Hearing loss was more than double in those with DS-AD (44%) compared to people with DS without AD [78]. Although there is no conclusive evidence to date that sensory disorders impair cognitive or social function in DS, it seems likely that such impairments result in withdrawal and lower engagement in everyday activities. Given this evidence, regularly evaluating vision and hearing could be an appropriate additional target for a multidomain non-pharmacological intervention.

#### Epilepsy

In adults with DS, epilepsy often occurs in later life and typically in association with dementia. This form of epilepsy was termed LOMEDS (late-onset myoclonic epilepsy in DS) and is associated with a more rapid decline of cognitive abilities in patients suffering from DS-AD [79]. Therefore, proactively screening for and treating epilepsy symptoms may significantly impact maintenance of cognitive performance, although is unlikely to affect the disease trajectory. More research is needed to understand the link between seizure risk and neuropathological processes in DS-AD.

### Limitations

The majority of included studies had small sample sizes, limiting statistical power and generalisability. Furthermore, all interventions to date have targeted single lifestyle domains; no multimodal intervention combining exercise, cognitive stimulation, and social engagement has been conducted in DS, representing a significant gap given the success of combined approaches in the general population. Publication bias is also a concern, as null findings from small studies may go unreported, potentially inflating cognitive effect sizes in the literature. Additionally, the high prevalence of co-occurring conditions in DS — including cardiovascular disease, thyroid dysfunction, diabetes, and sleep disorders — makes it difficult to isolate the independent contribution of any single lifestyle factor to cognition, and few studies adequately controlled for these confounders. Finally, while some studies used assessments developed for the general population, validated DS-specific cognitive instruments are increasingly available, including the mCRT [80] and the CAMCOG-DS-II [81], and should be prioritised in future research.

## Conclusion

Despite the fact that there are approximately 800,000 individuals with DS in the US and Europe alone [82, 83] with near-universal risk of DS-AD, there is a paucity of evidence on several key aspects. Specifically, there is limited information on the delivery, efficacy and potential modes of action of non-pharmacological interventions for dementia. Intervening at an early stage in the AD process could delay cognitive decline and maintain quality of life and level of independence. Given the increasing life expectancy of the approximately 6 million people with DS worldwide and the fact that AD is the main cause of mortality in DS, a global and interdisciplinary research effort is crucial.

## Supplementary Information

Supplementary Material 1.

Supplementary Material 2.

Supplementary Material 3.

## Data Availability

Not applicable.
