# Supplementary material for: Lifestyle intervention and cognitive outcomes in Down syndrome: a horizon 21 European Down syndrome consortium scoping review
Source: J Neurodev Disord. 2026 Apr 21;18:34. doi: 10.1186/s11689-026-09694-0 (PMC13231590; doi:10.1186/s11689-026-09694-0)
Supplement: Supplementary file 1 — Supplementary Material 1. [file 11689_2026_9694_MOESM1_ESM.docx]

**Protocol Strand 1: Exercise**

Concept 1: Down Syndrome

Medline: (MH "Down Syndrome")

EMBASE: 'Down syndrome'/exp

CINAHL (MH "Down Syndrome")

Web of Science : Keywords only on title and abstract

ASSIA: MAINSUBJECT.EXACT.EXPLODE("Down syndrome")

EBSCO Keywords: “down syndrome*” OR “downs syndrome*” OR “down disease” OR “Down's syndrome” OR “Downs syndrome” OR “langdon down disease” OR “mongol* idiocy” OR mongolism OR mongoloid OR “trisomy 21 syndrome” OR “Meiotic Nondisjunction”

EMBASE Keywords: “down syndrome*” OR “downs syndrome*” OR “down disease” OR “Down/s syndrome” OR “Downs syndrome” OR “langdon down disease” OR “mongol* idiocy” OR mongolism OR mongoloid OR “trisomy 21 syndrome” OR “Meiotic Nondisjunction”

Cognition:

Medline: (MH "Alzheimer Disease") OR (MH "Dementia+") OR (MH "Cognition+") OR (MH "Thinking+") OR (MH "Executive Function") OR (MH "Comprehension") OR (MH "Biomarkers+") OR (MH "Positron-Emission Tomography+") OR (MH "Magnetic Resonance Imaging+")

EMBASE: ('cognition'/exp OR 'mental capacity'/exp OR 'dementia'/exp OR 'biological marker'/exp OR 'positron emission tomography'/exp OR 'dementia'/exp OR 'biological marker'/exp OR 'positron emission tomography'/exp OR 'neuroimaging'/exp)

CINAHL: (MH "Dementia+") OR (MH "Alzheimer's Disease") OR (MH "Cognition") OR (MH "Imagination") OR (MH "Processing Speed") OR (MH "Executive Function") OR (MH "Perception") OR (MH "Thinking") OR (MH "Biological Markers+") OR (MH "Positron Emission Tomography Computed Tomography")

Web of Science : Keywords only on topic

ASSIA: MAINSUBJECT.EXACT.EXPLODE("Dementia") OR MAINSUBJECT.EXACT.EXPLODE("Cognition") OR MAINSUBJECT.EXACT.EXPLODE("Executive function") OR MAINSUBJECT.EXACT.EXPLODE("Alzheimers disease")

EBSCO cognition* OR cognitive* OR “mental capacity” OR ”mental abilit*” OR dementia* OR Alzheimer* OR dement* OR “mental competency” OR attention OR “executive function*” OR memory OR “mental perform*” OR thinking OR “mild cognitive impairment” OR prodromal OR “neurocognitive disorder*” OR “mental decline*” OR CAMCOG-DS OR “Severe Impairment Battery” OR “Test for Severe Impairment” OR R-BANS OR “Down syndrome mental status examination*” OR “Dementia questionnaire for people with learning disability*” OR “Vineland adaptive behaviour scale*” OR Biomarker* OR neuroimaging* OR Beta-amyloid OR amyloid-tau OR phospho-tau OR “neurofilament light chain” OR GFA OR CSF OR PET OR “positron emission tomography” OR MRI

Exercise:

Medline: (MH "Exercise+") OR (MH "Running+") OR (MH "Walking+") OR (MH "Physical Conditioning, Human+") (MH “Sports+”)

EMBASE: (‘exercise’/exp) OR ('sport'/exp)

CINAHL: (MH "Exercise+") OR (MH "Aerobic Exercises+") OR (MH "Muscle Strengthening+") OR (MH "Walking") OR (MH "Physical Fitness+") OR (MH "Physical Activity") OR (MH "Sports+") OR (MH "Physical Endurance+")

Web of Science : Keywords only on title and abstract

ASSIA: MAINSUBJECT.EXACT.EXPLODE("Water exercise") OR MAINSUBJECT.EXACT.EXPLODE("Cardiopulmonary exercise testing") OR MAINSUBJECT.EXACT.EXPLODE("Running") OR MAINSUBJECT.EXACT.EXPLODE("Walking") OR MAINSUBJECT.EXACT.EXPLODE("Dance exercise") OR MAINSUBJECT.EXACT.EXPLODE("Aerobic exercise") OR MAINSUBJECT.EXACT.EXPLODE("Exercise") OR MAINSUBJECT.EXACT.EXPLODE("Pelvic floor exercise") OR MAINSUBJECT.EXACT.EXPLODE("Exercise therapy") OR MAINSUBJECT.EXACT.EXPLODE("Sports")

EMBASE Keywords: exercis* OR walk* OR danc* OR swim* OR Jog* OR gym OR “physical fitness” OR running OR yoga OR pilates OR judo OR taekwando OR “marshal arts” OR “core strengthening” OR “circuit training” OR “getting active” OR “aqua fit” OR “strength training” OR “interval training” OR “weight lifting” OR “water sports” OR athletic* OR sport*

EBSCO: AB ( exercis* OR walk* OR danc* OR swim* OR Jog* OR gym* OR “physical fitness” OR running OR yoga OR pilates OR judo OR taekwando OR “marshal arts” OR “core strengthening” OR “circuit training” OR “getting active” OR “aqua fit” OR “strength training” OR “interval training” OR “weight lifting” OR “water sports” OR athletic* OR riding OR sport* ) OR TI ( exercis* OR walk* OR danc* OR swim* OR Jog* OR gym* OR “physical fitness” OR running OR yoga OR pilates OR judo OR taekwando OR “marshal arts” OR “core strengthening” OR “circuit training” OR “getting active” OR “aqua fit” OR “strength training” OR “interval training” OR “weight lifting” OR “water sports” OR athletic* OR riding OR sport*)

**Protocol Strand 2: Diet**

Concept 1: Down Syndrome

Medline: (MH "Down Syndrome")

EMBASE: 'Down syndrome'/exp

CINAHL (MH "Down Syndrome")

Web of Science : Keywords only on title and abstract

ASSIA: MAINSUBJECT.EXACT.EXPLODE("Down syndrome")

EBSCO Keywords: “down syndrome*” OR “downs syndrome*” OR “down disease” OR “Down's syndrome” OR “Downs syndrome” OR “langdon down disease” OR “mongol* idiocy” OR mongolism OR mongoloid OR “trisomy 21 syndrome” OR “Meiotic Nondisjunction”

EMBASE Keywords: “down syndrome*” OR “downs syndrome*” OR “down disease” OR “Down/s syndrome” OR “Downs syndrome” OR “langdon down disease” OR “mongol* idiocy” OR mongolism OR mongoloid OR “trisomy 21 syndrome” OR “Meiotic Nondisjunction”

Cognition:

Medline: (MH "Alzheimer Disease") OR (MH "Dementia+") OR (MH "Cognition+") OR (MH "Thinking+") OR (MH "Executive Function") OR (MH "Comprehension") OR (MH "Biomarkers+") OR (MH "Positron-Emission Tomography+") OR (MH "Magnetic Resonance Imaging+")

EMBASE: ('cognition'/exp OR 'mental capacity'/exp OR 'dementia'/exp OR 'biological marker'/exp OR 'positron emission tomography'/exp OR 'dementia'/exp OR 'biological marker'/exp OR 'positron emission tomography'/exp OR 'neuroimaging'/exp)

CINAHL: (MH "Dementia+") OR (MH "Alzheimer's Disease") OR (MH "Cognition") OR (MH "Imagination") OR (MH "Processing Speed") OR (MH "Executive Function") OR (MH "Perception") OR (MH "Thinking") OR (MH "Biological Markers+") OR (MH "Positron Emission Tomography Computed Tomography")

Web of Science : Keywords only on topic

ASSIA: MAINSUBJECT.EXACT.EXPLODE("Dementia") OR MAINSUBJECT.EXACT.EXPLODE("Cognition") OR MAINSUBJECT.EXACT.EXPLODE("Executive function") OR MAINSUBJECT.EXACT.EXPLODE("Alzheimers disease")

EBSCO cognition* OR cognitive* OR “mental capacity” OR ”mental abilit*” OR dementia* OR Alzheimer* OR dement* OR “mental competency” OR attention OR “executive function*” OR memory OR “mental perform*” OR thinking OR “mild cognitive impairment” OR prodromal OR “neurocognitive disorder*” OR “mental decline*” OR CAMCOG-DS OR “Severe Impairment Battery” OR “Test for Severe Impairment” OR R-BANS OR “Down syndrome mental status examination*” OR “Dementia questionnaire for people with learning disability*” OR “Vineland adaptive behaviour scale*” OR Biomarker* OR neuroimaging* OR Beta-amyloid OR amyloid-tau OR phospho-tau OR “neurofilament light chain” OR GFA OR CSF OR PET OR “positron emission tomography” OR MRI

Concept 3: Diet:

Medline: (MH "Diet, Food, and Nutrition+") OR (MH "Nutrients+") OR (MH "Fast Foods") OR (MH "Eating+") OR (MH "Healthy Lifestyle+")

EMBASE: 'diet'/exp OR 'nutrition'/exp OR 'Healthy Eating Index'/exp OR 'digestion'/exp OR 'eating'/exp

CINAHL (MH "Diet+") OR (MH "Nutrition+") OR (MH "Access to Healthy Foods") OR (MH "Food Habits")

Web of Science : Keywords only on title and abstract

ASSIA: MAINSUBJECT.EXACT.EXPLODE("Fast food") OR MAINSUBJECT.EXACT.EXPLODE("Food consumption") OR MAINSUBJECT.EXACT.EXPLODE("Food supplements") OR MAINSUBJECT.EXACT.EXPLODE("Diet") OR MAINSUBJECT.EXACT.EXPLODE("Food") OR MAINSUBJECT.EXACT.EXPLODE("Food habits") OR MAINSUBJECT.EXACT.EXPLODE("Nutrition") OR MAINSUBJECT.EXACT("Dietary fibre") OR MAINSUBJECT.EXACT.EXPLODE("Healthy food")

EMBASE Keywords: diet* OR nutrition* OR nutrient* OR food* OR protein* OR carbohydrate* OR eating OR digestion OR digestive OR feeding OR macronutient* OR vitamin* OR mineral* OR “meal replacement*” OR “meal planning*” OR “healthy eating” OR vegetable* OR “water intake” OR consumption OR fibre* OR “healthy gut*”

**Protocol Strand 3 Social Connectedness**

Concept 1: Down Syndrome

Medline: (MH "Down Syndrome")

EMBASE: 'Down syndrome'/exp

CINAHL (MH "Down Syndrome")

Web of Science : Keywords only on title and abstract

ASSIA: MAINSUBJECT.EXACT.EXPLODE("Down syndrome")

EBSCO Keywords: “down syndrome*” OR “downs syndrome*” OR “down disease” OR “Down's syndrome” OR “Downs syndrome” OR “langdon down disease” OR “mongol* idiocy” OR mongolism OR mongoloid OR “trisomy 21 syndrome” OR “Meiotic Nondisjunction”

EMBASE Keywords: “down syndrome*” OR “downs syndrome*” OR “down disease” OR “Down's syndrome” OR “Downs syndrome” OR “langdon down disease” OR “mongol* idiocy” OR mongolism OR mongoloid OR “trisomy 21 syndrome” OR “Meiotic Nondisjunction”

Cognition:

Medline: (MH "Alzheimer Disease") OR (MH "Dementia+") OR (MH "Cognition+") OR (MH "Thinking+") OR (MH "Executive Function") OR (MH "Comprehension") OR (MH "Biomarkers+") OR (MH "Positron-Emission Tomography+") OR (MH "Magnetic Resonance Imaging+")

EMBASE: ('cognition'/exp OR 'mental capacity'/exp OR 'dementia'/exp OR 'biological marker'/exp OR 'positron emission tomography'/exp OR 'dementia'/exp OR 'biological marker'/exp OR 'positron emission tomography'/exp OR 'neuroimaging'/exp)

CINAHL: (MH "Dementia+") OR (MH "Alzheimer's Disease") OR (MH "Cognition") OR (MH "Imagination") OR (MH "Processing Speed") OR (MH "Executive Function") OR (MH "Perception") OR (MH "Thinking") OR (MH "Biological Markers+") OR (MH "Positron Emission Tomography Computed Tomography")

Web of Science : Keywords only on topic

ASSIA: MAINSUBJECT.EXACT.EXPLODE("Dementia") OR MAINSUBJECT.EXACT.EXPLODE("Cognition") OR MAINSUBJECT.EXACT.EXPLODE("Executive function") OR MAINSUBJECT.EXACT.EXPLODE("Alzheimers disease")

EBSCO cognition* OR cognitive* OR “mental capacity” OR ”mental abilit*” OR dementia* OR Alzheimer* OR dement* OR “mental competency” OR attention OR “executive function*” OR memory OR “mental perform*” OR thinking OR “mild cognitive impairment” OR prodromal OR “neurocognitive disorder*” OR “mental decline*” OR CAMCOG-DS OR “Severe Impairment Battery” OR “Test for Severe Impairment” OR R-BANS OR “Down syndrome mental status examination*” OR “Dementia questionnaire for people with learning disability*” OR “Vineland adaptive behaviour scale*” OR Biomarker* OR neuroimaging* OR Beta-amyloid OR amyloid-tau OR phospho-tau OR “neurofilament light chain” OR GFA OR CSF OR PET OR “positron emission tomography” OR MRI

Concept 3: Social Connectedness

Medline: (MH "Loneliness") OR (MH "Social Isolation+") OR (MH "Social Alienation") OR (MH "Social Support+") OR (MH "Social Environment+") OR (MH "Social Participation") OR (MH "Social Interaction") OR (MH "Social Group")

CINAHL/; (MH "Loneliness") OR (MH "Social Isolation+") OR (MH "Social Alienation") OR (MH "Friendship") OR (MH "Social Networks")

EMBASE: 'loneliness'/exp OR 'social exclusion'/de OR 'connectedness'/exp OR 'friendship'/exp OR 'social network'/exp

Web of Science

ASSIA: MAINSUBJECT.EXACT("Close friendships") OR MAINSUBJECT.EXACT.EXPLODE("Intimate relationships") OR MAINSUBJECT.EXACT.EXPLODE("Social networks") OR MAINSUBJECT.EXACT.EXPLODE("Friendships") OR MAINSUBJECT.EXACT.EXPLODE("Loneliness") OR MAINSUBJECT.EXACT.EXPLODE("Social exclusion")

EMBASE Keywords: connect* OR social* OR kinship OR relationship* OR friend* OR ally OR allied OR unity OR community* OR “support group” OR lover* OR partner* OR spous* OR girlfriend* OR boyfriend* OR husband* OR wife* OR alone* OR isolat* OR exclude* OR abandon* OR lonely OR loneliness OR belong* OR "famil* environ*" OR "famil* setting*" OR "home setting" OR "home environment" OR recreation* OR alienat*

**Protocol Strand 4: Cognitive Stimulation**

Concept 1: Down Syndrome

Medline: (MH "Down Syndrome")

EMBASE: 'Down syndrome'/exp

CINAHL (MH "Down Syndrome")

Web of Science : Keywords only on title and abstract

ASSIA: MAINSUBJECT.EXACT.EXPLODE("Down syndrome")

EBSCO Keywords: “down syndrome*” OR “downs syndrome*” OR “down disease” OR “Down's syndrome” OR “Downs syndrome” OR “langdon down disease” OR “mongol* idiocy” OR mongolism OR mongoloid OR “trisomy 21 syndrome” OR “Meiotic Nondisjunction”

EMBASE Keywords: “down syndrome*” OR “downs syndrome*” OR “down disease” OR “Down's syndrome” OR “Downs syndrome” OR “langdon down disease” OR “mongol* idiocy” OR mongolism OR mongoloid OR “trisomy 21 syndrome” OR “Meiotic Nondisjunction”

Cognition:

Medline: (MH "Alzheimer Disease") OR (MH "Dementia+") OR (MH "Cognition+") OR (MH "Thinking+") OR (MH "Executive Function") OR (MH "Comprehension") OR (MH "Biomarkers+") OR (MH "Positron-Emission Tomography+") OR (MH "Magnetic Resonance Imaging+")

EMBASE: ('cognition'/exp OR 'mental capacity'/exp OR 'dementia'/exp OR 'biological marker'/exp OR 'positron emission tomography'/exp OR 'dementia'/exp OR 'biological marker'/exp OR 'positron emission tomography'/exp OR 'neuroimaging'/exp)

CINAHL: (MH "Dementia+") OR (MH "Alzheimer's Disease") OR (MH "Cognition") OR (MH "Imagination") OR (MH "Processing Speed") OR (MH "Executive Function") OR (MH "Perception") OR (MH "Thinking") OR (MH "Biological Markers+") OR (MH "Positron Emission Tomography Computed Tomography")

Web of Science : Keywords only on topic

ASSIA: MAINSUBJECT.EXACT.EXPLODE("Dementia") OR MAINSUBJECT.EXACT.EXPLODE("Cognition") OR MAINSUBJECT.EXACT.EXPLODE("Executive function") OR MAINSUBJECT.EXACT.EXPLODE("Alzheimers disease")

EBSCO cognition* OR cognitive* OR “mental capacity” OR ”mental abilit*” OR dementia* OR Alzheimer* OR dement* OR “mental competency” OR attention OR “executive function*” OR memory OR “mental perform*” OR thinking OR “mild cognitive impairment” OR prodromal OR “neurocognitive disorder*” OR “mental decline*” OR CAMCOG-DS OR “Severe Impairment Battery” OR “Test for Severe Impairment” OR R-BANS OR “Down syndrome mental status examination*” OR “Dementia questionnaire for people with learning disability*” OR “Vineland adaptive behaviour scale*” OR Biomarker* OR neuroimaging* OR Beta-amyloid OR amyloid-tau OR phospho-tau OR “neurofilament light chain” OR GFA OR CSF OR PET OR “positron emission tomography” OR MRI

Concept 3: Cognitive Stimulation

Medline: (MH "Cognitive Training")  OR (MH "Games, Recreational") OR (MH "Video Games+")

EMBASE: 'cognitive stimulation'/exp OR 'cognitive rehabilitation'/exp OR 'recreational game'/exp

CINAHL: (MH "Games+") OR (MH "Video Games+")

Web of Science : Keywords only on title and abstract

ASSIA:

EMBASE Keywords: “Cognitive stimulat*” OR “mental stimulat*” OR “cognitive train*” OR “cognitive exercise*” OR “brain train*” OR puzzle* OR sudoku OR “brain game*” OR “brain teaser*” OR crossword* OR “word search*” OR “educational game*” OR “virtual game*” OR “video game*” OR “interactive game*” OR “games educat*” OR game*based OR “memory game*” OR gamification*

**Protocol Strand 5: Cardiovascular**

Concept 1: Down Syndrome

Medline: (MH "Down Syndrome")

EMBASE: 'Down syndrome'/exp

CINAHL (MH "Down Syndrome")

Web of Science : Keywords only on title and abstract

ASSIA: MAINSUBJECT.EXACT.EXPLODE("Down syndrome")

EBSCO Keywords: “down syndrome*” OR “downs syndrome*” OR “down disease” OR “Down's syndrome” OR “Downs syndrome” OR “langdon down disease” OR “mongol* idiocy” OR mongolism OR mongoloid OR “trisomy 21 syndrome” OR “Meiotic Nondisjunction”

EMBASE Keywords: “down syndrome*” OR “downs syndrome*” OR “down disease” OR “Down's syndrome” OR “Downs syndrome” OR “langdon down disease” OR “mongol* idiocy” OR mongolism OR mongoloid OR “trisomy 21 syndrome” OR “Meiotic Nondisjunction”

Cognition:

Medline: (MH "Alzheimer Disease") OR (MH "Dementia+") OR (MH "Cognition+") OR (MH "Thinking+") OR (MH "Executive Function") OR (MH "Comprehension") OR (MH "Biomarkers+") OR (MH "Positron-Emission Tomography+") OR (MH "Magnetic Resonance Imaging+")

EMBASE: ('cognition'/exp OR 'mental capacity'/exp OR 'dementia'/exp OR 'biological marker'/exp OR 'positron emission tomography'/exp OR 'dementia'/exp OR 'biological marker'/exp OR 'positron emission tomography'/exp OR 'neuroimaging'/exp)

CINAHL: (MH "Dementia+") OR (MH "Alzheimer's Disease") OR (MH "Cognition") OR (MH "Imagination") OR (MH "Processing Speed") OR (MH "Executive Function") OR (MH "Perception") OR (MH "Thinking") OR (MH "Biological Markers+") OR (MH "Positron Emission Tomography Computed Tomography")

Web of Science : Keywords only on topic

ASSIA: MAINSUBJECT.EXACT.EXPLODE("Dementia") OR MAINSUBJECT.EXACT.EXPLODE("Cognition") OR MAINSUBJECT.EXACT.EXPLODE("Executive function") OR MAINSUBJECT.EXACT.EXPLODE("Alzheimers disease")

EBSCO cognition* OR cognitive* OR “mental capacity” OR ”mental abilit*” OR dementia* OR Alzheimer* OR dement* OR “mental competency” OR attention OR “executive function*” OR memory OR “mental perform*” OR thinking OR “mild cognitive impairment” OR prodromal OR “neurocognitive disorder*” OR “mental decline*” OR CAMCOG-DS OR “Severe Impairment Battery” OR “Test for Severe Impairment” OR R-BANS OR “Down syndrome mental status examination*” OR “Dementia questionnaire for people with learning disability*” OR “Vineland adaptive behaviour scale*” OR Biomarker* OR neuroimaging* OR Beta-amyloid OR amyloid-tau OR phospho-tau OR “neurofilament light chain” OR GFA OR CSF OR PET OR “positron emission tomography” OR MRI

Concept 3: Cardiovascular

Medline: (MH "Heart Disease Risk Factors+") OR (MH "Cardiovascular System+") OR (MH "Cardiovascular Abnormalities+") OR (MH "Cardiovascular Diseases+") OR (MH "Body Weight+") OR (MH "Adipose Tissue+") OR (MH "Obesity+") OR (MH "Pediatric Obesity") OR (MH "Diabetes Mellitus+") OR (MH "Myocardial Infarction+")

EMBASE: 'cardiovascular system'/exp OR 'obesity'/exp OR 'heart'/exp OR 'blood pressure'/exp OR 'hypertension'/exp OR 'cholesterol'/exp OR 'hydroxymethylglutaryl coenzyme A reductase inhibitor'/exp OR 'body mass'/exp OR 'diabetes mellitus'/exp

CINAHL: (MH "Coronary Disease+") OR (MH "Myocardial Ischemia+") OR (MH "Heart Diseases+") OR (MH "Pulmonary Heart Disease") OR (MH "Heart Valve Diseases+") OR (MH "Blood Pressure+") OR (MH "Hypertension+") OR (MH "Hypotension+") OR (MH "Obesity+") OR (MH "Diabetes Mellitus+")

Web of Science : Keywords only on title and abstract

ASSIA: MAINSUBJECT.EXACT.EXPLODE("Diabetes") OR MAINSUBJECT.EXACT.EXPLODE("Cardiovascular diseases") OR MAINSUBJECT.EXACT.EXPLODE("Congenital heart disease") OR MAINSUBJECT.EXACT.EXPLODE("Cardiovascular system") OR MAINSUBJECT.EXACT.EXPLODE("Body weight") OR MAINSUBJECT.EXACT("Cardiology") OR MAINSUBJECT.EXACT.EXPLODE("Heart arrhythmia") OR MAINSUBJECT.EXACT.EXPLODE("Blood pressure") OR MAINSUBJECT.EXACT.EXPLODE("Cholesterol") OR MAINSUBJECT.EXACT.EXPLODE("Heart failure") OR MAINSUBJECT.EXACT("Cardiopulmonary diseases") OR MAINSUBJECT.EXACT.EXPLODE("Weight gain") OR MAINSUBJECT.EXACT.EXPLODE("Heart") OR MAINSUBJECT.EXACT.EXPLODE("Body mass") OR MAINSUBJECT.EXACT.EXPLODE("Weight related disorders") OR MAINSUBJECT.EXACT.EXPLODE("Hypotension") OR MAINSUBJECT.EXACT.EXPLODE("Heart attacks") OR MAINSUBJECT.EXACT.EXPLODE("Heart diseases") OR MAINSUBJECT.EXACT("Cardiovascular response")
