# Supplementary material for: Lifestyle intervention and cognitive outcomes in Down syndrome: a horizon 21 European Down syndrome consortium scoping review
Source: J Neurodev Disord. 2026 Apr 21;18:34. doi: 10.1186/s11689-026-09694-0 (PMC13231590; doi:10.1186/s11689-026-09694-0)
Supplement: Supplementary file 2 — Supplementary Material 2. [file 11689_2026_9694_MOESM2_ESM.docx]

| **Supplementary Table 1 : Inclusion and Exclusion Criteria Applied to Articles identified in Literature Search** | | |
| --- | --- | --- |
|  | **Inclusion** | **Exclusion** |
| **Population** | Adults (18+) with Down syndrome | all participants younger than 18 years  Cause of ID other than Down syndrome |
| **Concept** | Studies reporting cognitive/biomarkers outcomes.  Studies where multiple outcomes are reported but cognitive outcomes must be reported separately from other outcomes.  Relationship between domain of interest and cognitive/biomarker outcomes. | No cognitive/or biomarker related outcomes reported.  No domain of interest included |
| **Context** | Intervention studies  Randomized Control Trials  Observational studies (retrospective and cross-sectional)  Cohort studies  Longitudinal studies | Opinion papers, Editorials, Conference abstracts, Case Reports  Systematic reviews, meta-analyses, narrative reviews, critical reviews, qualitative reviews, Theses/dissertations |
