# Supplementary material for: Lifestyle intervention and cognitive outcomes in Down syndrome: a horizon 21 European Down syndrome consortium scoping review
Source: J Neurodev Disord. 2026 Apr 21;18:34. doi: 10.1186/s11689-026-09694-0 (PMC13231590; doi:10.1186/s11689-026-09694-0)
Supplement: Supplementary file 3 — Supplementary Material 3. [file 11689_2026_9694_MOESM3_ESM.docx]

**Strand 1: Exercise**

**Identification**

References from other sources **(n = )**

Citation searching (n = )

Grey literature (n = )

Studies screened **(n = 1060)**

Studies sought for retrieval **(n = 91)**

Studies assessed for eligibility **(n = 91)**

References removed **(n = 307)**

Duplicates identified manually (n = 4)

Duplicates identified by Covidence (n = 303)

Marked as ineligible by automation tools (n = 0)

Other reasons (n = )

Studies excluded **(n = 622)**

Studies not retrieved **(n = 0)**

Studies excluded **(n = 71)**

not DS (n = 10)

duplicate (n = 3)

review paper (n = 11)

Paediatric population (n = 19)

Not related to cognition (n = 14)

Can't separate DS from other ID (n = 3)

Does not include exercise component (n = 11)

**Included**

Studies included in review **(n = 20)**

**Screening**

Studies from databases/registers **(n = 1313)**

Embase (n = 597)

Web of Science (n = 345)

MEDLINE (n = 211)

CINAHL (n = 103)

ASSIA (n = 57)

**Strand 2: Diet**

**Identification**

References from other sources **(n = )**

Citation searching (n = )

Grey literature (n = )

Studies screened **(n = 6501)**

Studies sought for retrieval **(n = 36)**

Studies assessed for eligibility **(n = 36)**

References removed **(n = 3096)**

Duplicates identified manually (n = 10)

Duplicates identified by Covidence (n = 3086)

Marked as ineligible by automation tools (n = 0)

Other reasons (n = )

Studies excluded **(n = 6465)**

Studies not retrieved **(n = 0)**

Studies excluded **(n = 33)**

Wrong study type (n = 5)

not related to DS (n = 3)

Not related to diet (n = 8)

supplements not diet (n = 6)

not related to cognition (n = 14)

**Included**

Studies included in review **(n = 0)**

**Screening**

Studies from databases/registers **(n = 9597)**

Web of Science (n = 4096)

Embase (n = 3207)

MEDLINE (n = 1987)

CINAHL (n = 246)

ASSIA (n = 61)

**Strand 3: Social Connectedness**

**Identification**

**Included**

**Screening**

Studies from databases/registers **(n = 9312)**

Embase (n = 3733)

Web of Science (n = 2334)

MEDLINE (n = 1794)

ASSIA (n = 932)

CINAHL (n = 492)

Google Scholar (n = 27)

References from other sources **(n = )**

Citation searching (n = )

Grey literature (n = )

Studies included in review **(n = 6)**

Studies excluded **(n = 5970)**

Studies not retrieved **(n = 0)**

Studies assessed for eligibility **(n = 48)**

Studies sought for retrieval **(n = 48)**

Studies screened **(n = 6018)**

References removed **(n = 2767)**

Duplicates identified manually (n = 0)

Duplicates identified by Covidence (n = 2767)

Marked as ineligible by automation tools (n = 0)

Other reasons (n = )

Studies excluded **(n = 42)**

review paper (n = 1)

Wrong outcomes (n = 30)

Wrong comparator (n = 1)

Wrong indication (n = 2)

Paediatric population (n = 3)

Wrong patient population (n = 5)

**Strand 4: Cognitive Stimulation**

**Identification**

**Included**

**Screening**

References from other sources **(n = )**

Citation searching (n = )

Grey literature (n = )

Studies screened **(n = 145)**

Studies sought for retrieval **(n = 20)**

Studies assessed for eligibility **(n = 20)**

References removed **(n =81)**

Duplicates identified manually (n = 1)

Duplicates identified by Covidence (n = 80)

Marked as ineligible by automation tools (n = 0)

Other reasons (n = )

Studies excluded **(n = 125)**

Studies not retrieved **(n = 0)**

Studies excluded **(n = 16)**

duplicate (n = 1)

not cognitive (n = 2)

review paper (n = 4)

Wrong intervention (n = 1)

conference abstract (n = 1)

not cognitive stimulation (n = 1)

protocol paper no results (n = 3)

could not separate DS from ID (n = 3)

Studies included in review **(n = 4)**

Studies from databases/registers **(n = 226)**

Web of Science (n = 66)

Embase (n = 75

MEDLINE (n = 28)

CINAHL (n = 16)

ASSIA (n = 10)

**Strand 5: Cardiovascular**

**Identification**

References from other sources **(n = )**

Citation searching (n = )

Grey literature (n = )

Studies screened **(n = 3144)**

Studies sought for retrieval **(n = 74)**

Studies assessed for eligibility **(n = 74)**

References removed **(n = 1182)**

Duplicates identified manually (n = 20)

Duplicates identified by Covidence (n = 1182)

Marked as ineligible by automation tools (n = 0)

Other reasons (n = )

Studies excluded **(n = 3070)**

Studies not retrieved **(n = 0)**

Studies excluded **(n = 60)**

Review paper (n = 5)

Wrong outcomes (n = 6)

Wrong intervention (n = 11)

conference abstract (n = 2)

Paediatric population (n = 13)

not related to cardio (n = 7)

Not DS (n = 5)

not related to cognition (n = 11)

**Included**

Studies included in review **(n = 14)**

**Screening**

Studies from databases/registers **(n = 4326)**

Embase (n = 2334)

MEDLINE (n = 1155)

Web of Science (n = 807)

CINAHL (n = 263)

ASSIA (n = 65)
